# Supplementary material for: Comparison of impact accelerations between injury-resistant and recently injured recreational runners
Source: PLoS One. 2022 Sep 9;17(9):e0273716. doi: 10.1371/journal.pone.0273716 (PMC9462674; doi:10.1371/journal.pone.0273716)
Supplement: Attachment — Submitted filename: Response to Reviewers.docx [file pone.0273716.s002.docx]

| Section | Line Number | Reviewer Comments | Changes Made | Revised Line Number | Author Comments |
| --- | --- | --- | --- | --- | --- |
| General | N/A | “Please ensure that your manuscript meets PLOS ONE's style requirements, including those for file naming. The PLOS ONE style templates can be found at  <https://journals.plos.org/plosone/s/file?id=wjVg/PLOSOne_formatting_sample_main_body.pdf> and  <https://journals.plos.org/plosone/s/file?id=ba62/PLOSOne_formatting_sample_title_authors_affiliations.pdf>” | Headings, sub-headings, Figures and Tables have been amended to specifications outlined in online PDF | N/A | N/A |
| References | N/A | “Please review your reference list to ensure that it is complete and correct. If you have cited papers that have been retracted, please include the rationale for doing so in the manuscript text, or remove these references and replace them with relevant current references. Any changes to the reference list should be mentioned in the rebuttal letter that accompanies your revised manuscript. If you need to cite a retracted article, indicate the article’s retracted status in the References list and also include a citation and full reference for the retraction notice.” | One reference added following suggestion of Reviewer 2. This reference is Milner et al., 2006 [3]. | N/A | N/A |
| Acknowledgements | 344-347 | “We note that you have provided funding information that is not currently declared in your Funding Statement. However, funding information should not appear in the Acknowledgments section or other areas of your manuscript. We will only publish funding information present in the Funding Statement section of the online submission form.  Please remove any funding-related text from the manuscript and let us know how you would like to update your Funding Statement. Currently, your Funding Statement reads as follows:  "This study has emanated from research supported by Science Foundation Ireland (SFI) under grant number SFI/12/RC/2289_P2, cofounded by the European Regional Development Fund. The authors thank the participants for their time in partaking in this study.”” | Removed “This study has emanated from research supported by Science Foundation Ireland (SFI) under grant number SFI/12/RC/2289_P2, cofounded by the European Regional Development Fund.” from Acknowledgements section | 375-377 | Thank you for noting this. The funding declaration has been removed from the Acknowledgements section |
| Competing Interests | 351-354 | “Thank you for stating the following in your Competing Interests section:  "The authors declare that they have no competing interests. The results of the study are presented clearly, honestly, and without fabrication, falsification, or inappropriate data manipulation."  Please complete your Competing Interests on the online submission form to state any Competing Interests. If you have no competing interests, please state "The authors have declared that no competing interests exist.", as detailed online in our guide for authors at <http://journals.plos.org/plosone/s/submit-now>   This information should be included in your cover letter; we will change the online submission form on your behalf.” | Removed “The authors declare that they have no competing interests. The results of the study are presented clearly, honestly, and without fabrication, falsification, or inappropriate data manipulation.” from Competing Interests section. Instead states “The authors have declared that no competing interests exist.” | 383 - 384 | Competing interests statement amended as recommended |
|  |  | “We note that you have indicated that data from this study are available upon request. PLOS only allows data to be available upon request if there are legal or ethical restrictions on sharing data publicly. For more information on unacceptable data access restrictions, please see <http://journals.plos.org/plosone/s/data-availability#loc-unacceptable-data-access-restrictions>.  In your revised cover letter, please address the following prompts:  a) If there are ethical or legal restrictions on sharing a de-identified data set, please explain them in detail (e.g., data contain potentially sensitive information, data are owned by a third-party organization, etc.) and who has imposed them (e.g., an ethics committee). Please also provide contact information for a data access committee, ethics committee, or other institutional body to which data requests may be sent.  b) If there are no restrictions, please upload the minimal anonymized data set necessary to replicate your study findings as either Supporting Information files or to a stable, public repository and provide us with the relevant URLs, DOIs, or accession numbers. For a list of acceptable repositories, please see <http://journals.plos.org/plosone/s/data-availability#loc-recommended-repositories>.  We will update your Data Availability statement on your behalf to reflect the information you provide.” | - | N/A | Data available upon request was indicated in error, and the authors express our apologies. Sharing of data was not part of explicit consent within the ethics application and informed consent form, and therefore, due to GDPR constraints in Europe, we cannot share the dataset. |
|  |  | Reviewer #1 “Participants were recruited from online survey, and how does one ensure participants were well versed with the questions from the survey especially those with medical terms (for injury) to reduce biased and error?” | Inserted “Prior to any physical testing, the primary researchers checked the survey responses for accuracy and completion, with all injury and training behaviour responses clarified with participants.” Into Procedures sub-section. | 146 - 148 | Injury was defined as stated in Lines 127-131.  A sentence has also been added to the procedures section to reflect the measures taken to clarify the accuracy and understanding of the survey and responses given. |
|  |  | Reviewer #1 “The paper compares the impact of accelerations in runners with history of any overuse RRI and not specific to injury locations directly, does this effect the overall data other than mentioned in L291 – 298?” | - | N/A | We’re not exactly sure what you mean by affecting the overall data – however we do not think so, beyond the limitation stated in lines 352-353.  We did not have enough quantities of specific RRIs to examine the effects of impact acceleration on particular pathologies, so the analysis would have been underpowered. Also, an advantage of looking at general RRIs is if you want to develop injury prevention programmes, they are typically developed for all RRIs and not specific pathologies per se, thus giving the current analysis a degree of ecological validity. |
| General | N/A | Reviewer #2 “The reviewer understands how authors categorized into three groups. Based on the findings, how would the authors translate the results for the runners who had injuries in the past within 1-2 years? I mean, this group is missing in the analyses. Is there any specific reason why this group has not been included in the analyses?” | - | N/A | As stated in the manuscript, “runners who had been injured 1-2 years pre-testing were excluded from selection in order to ensure a clear demarcation between the “injury resistant” and “recently injured” running groups (Dillon et al., 2021).”  It is likely that these runners will ultimately enter into the injury resistant group or the recently injured group over time, but at the time of analysis, these runners were in a middle ground that may have clouded the results. Therefore, authors believed it may be most appropriate to remove these runners from analysis. |
| Introduction | 84-86 | Reviewer #2 “Lines 84-86: Authors stated, “However, impact accelerations at the sacrum have rarely been assessed despite the prevalence of lower back and hip injuries experienced in runners.” Why is that?“ | - | N/A | Authors are unsure why this is. Tibia/lower leg injures are certainly more prevalent than lower back/hip injuries within this population, so that has perhaps inspired so many researchers to investigate loading that is local to this site. In addition, impact accelerometers have been found to be reliable measures of loading at the tibia/shank, but less research has investigated the reliability of these devices at the sacrum. This may influence the methods of research in this area.  Authors would be happy to add a sentence explaining this if reviewers feel it would be beneficial. |
| Methods | 140 | Reviewer #2 “Line 140: Take out the line “Participants completed a Physical Activity Readiness Questionnaire (PAR-Q)” unless the authors report anything from this questionnaire, at least in this manuscript.“ | Removed “completed a Physical Activity Readiness Questionnaire (PAR-Q) and” | 146 | PAR-Q statement removed as advised. |
| Methods | 162-163 | Reviewer #2 “Lines 162-163: What was the actual treadmill running time? The authors stated 6 minutes but mentioned that this was familiarization with treadmill running. Why were specific 6 minutes chosen for familiarization?” | Participants ran on the treadmill for a mean time of 12 minutes and 32 seconds (± 5 minutes and 31 seconds). | 222-223 | 6 minutes was chosen as this is the amount of time that has been found previously (Lavcanska 2005) for runners to become familiar with treadmill running. Kinematic parameters had been assessed from the start to the end of the treadmill run in this study, but parameters did not change following the 6 minute mark.  Mean and SD running times have been added to the manuscript for clarity. |
| Methods | 171-172 | Reviewer #2 “Lines 170-171: Peakaccel was taken from the initial contact. Is it for both the tibia and sacrum? Why was initial contact chosen to identify the peak value?“ | - | N/A | “Peakaccel was taken as the maximal amplitude of the accelerometer’s local maxima at initial contact” – this is the point when the maximal acceleration has occurred, and as a result, we feel is appropriately described as the peak. |
| Methods | Figure 1 | Reviewer #2 “Figure 1 legend is confusing. Do the left and right figures mean for shank and sacrum? Then please fix (A) and (B) explanations accordingly.“ | Amended Figure 1 for clarity | N/A | Amended Figure 1 so that shank and sacrum are more clearly indicated |
| Methods | 188-190 | Reviewer #2 “Lines 188-190: Give some references to remove the outliers based on the condition that the values are beyond 1.5 times of upper and lower quartiles.“ | Reference for Milner et al., 2006 added | 200 | Reference for Milner et al., 2006 added |
| Methods | 201-202 | Reviewer #2 “Lines 201-202: Please mention the specific references based on what effect size criteria were chosen, such as trivial effect size = 0-0.19, small effect size = 0.2-.59. Are these chosen based on reference 37?” | Added a ; in sentence to indicate a follow on from Cohen (37) | 210-212 | Yes, these effect sizes are based on Cohen (reference 37) |
| Results | 206-207 | Reviewer #2 “Lines 206-207: The reviewer is unsure how 147 subjects were chosen from a total of 310 subjects. The reviewer acknowledges the criteria for selecting the group as injury types, sex, quarterly annual mileage, and typical training speed; did only these 147 subjects match these criteria above out of 310 or more? Is there any bias when selecting 49 subjects for each group?” | Statement on selection process added to methods to state “Where more than one recently injured or acquired injury resistant runner could be matched to the never injured runner, the runner was chosen at random by flipping a coin, so as to eliminate bias from the matching selection.” | 139 - 142 | The never injured group (n = 49) were the primary reference group for matching. Runners who had been recently injured were then matched by sex, quarterly annual mileage and typical training speed to the 49 never injured runners – where more than one runner could be a potential match, the runner was chosen at random by flipping a coin. This was done for the acquired injury resistance group too, so as to eliminate bias from the matching selection. |
| Results | Table 2 | Reviewer #2 “Table 2 has not been explained in the Results section. The authors only stated it in lines 211-212. Also, table 2 says that there were 86 injuries among 49 runners. Considering the breakdown for RRI, 36*1 + 11*2 + 1*3 + 1*4 = 65. The reviewer is unsure how the authors got 86 injures for 49 runners, or the reviewer is missing something here. Clarify this.” | Sentence added stating “The knee was the most commonly injured region (23%), followed by the calf (20% and foot (15%).  Table 2 had an error – 65 injuries is the correct number of injuries – Table 2 has been amended. | 224-225, 234 | Thank you for highlighting this error – the table has been amended with accurate information regarding RRI location. |
| Discussion | 254-300 | Reviewer #2 “Discussion: 1st paragraph, lines 250-285, is a long one. Divide into multiple sections.” | Break in paragraph at line 267-268 | 279-280 | Paragraph separated to form multiple sections. |
| Discussion | 254 | Reviewer #2 “Please include hypothesis 1 in line 254. During the last paragraph of the introduction, the authors mentioned their primary hypothesis, but nothing was mentioned in the discussion. Include hypothesis around line 254, where authors stated the primary findings, then go for the discussion based on the findings and relate the findings to the hypothesis.” | Hypothesis support and rejection stated throughout the discussion more clearly | 265-270, 313, 328 | Thank you for this suggestion. Manuscript has been amended accordingly. |
| Conclusion | 344 | Reviewer #2 “Conclusion: Lines 330-342: I would concise the two paragraphs into one paragraph and make it a little short.” | Removed some aspects of the conclusion to condense findings into one smaller paragraph | 357-373 | Agreed, the paragraph was quite long and maybe not concise enough. Efforts have been made to amend this. |
|  |  | Reviewer #2 “Since the authors main findings suggest that Rateaccel at the sacrum was higher in recently injured runners. Does this mean that they are more likely to be injured than the other two groups? If not, please explain why? If yes, the reviewer is curious about the potential solutions/rehabilitation thoughts the authors would likely suggest for recently injured runners based on that they have greater Rateaccel at the sacrum.” | - | N/A | As the Rateaccel at the sacrum was found to be higher in recently injured runners, it does suggest that this component may link to injury yes (or at least in the short-term). With this in mind, an injury rehabilitation or running re-training intervention programme may be warranted for those who have higher rates of acceleration at the sacrum. Examples of gait retraining for impact acceleration attenuation have been observed through the literature (Crowell, 2010; Wood, 2014; Bowser, 2018), and have proven to be effective in reducing impact loading in injured and uninjured populations.  Authors would happily expand on this in the conclusion or end of discussion if the reviewer felt it was warranted. |
| Conclusion | 338-342 | Reviewer #2 “Lines 338-342: The reviewer is unclear about what the authors want to say here. Please rephrase. The terms ‘shock absorption’ and ‘gait re-education’ have not been discussed in the manuscript and are suddenly thrown in conclusion to end the manuscript.” | Replaced shock absorption with impact attenuation for clarity. Also indicated the use of gait re-education for impact attenuation and potential injury prevention. | 364, 373 | Thank you for this valid point. Shock absorption was a poor choice of phraseology for this article, given that it had not been mentioned previously. Phrase amended to state impact attenuation instead, which hopefully sits better within the article. |
