# Supplementary material for: Comparison of impact accelerations between injury-resistant and recently injured recreational runners
Source: PLoS One. 2022 Sep 9;17(9):e0273716. doi: 10.1371/journal.pone.0273716 (PMC9462674; doi:10.1371/journal.pone.0273716)
Supplement: Attachment — Submitted filename: Response to Reviewers.docx [file pone.0273716.s004.docx]

| Section | Line Number | Reviewer #2 Comments | Changes Made | Revised Line Number | Author Comments |
| --- | --- | --- | --- | --- | --- |
| Introduction | 84-86 (Original manuscript) | Authors have answered the question regarding their views. It would be beneficial to add their thoughts to the revised manuscript. | “As impact accelerometers have been found to be reliable measures of loading [28], particularly with reference to the magnitude of loading at the tibia, this may have influenced the choice of methods in studies investigating the relationship between impact acceleration and RRIs. More recently, the magnitude and rate of acceleration at both the tibia and sacrum have been found to be acceptable for injury-related research [29], and so the examination of both segments is warranted.” | 89-94 | Suggested changes have been added to manuscript. |
| Methods | 175-176 | This is still not clear what are the protocols for actual treadmill running. The authors added the lines in the Results section instead of the methods section without describing the protocol. | “Following the 6 minutes of familiarisation, the participants continued to run at 9km/hr for an additional 1 minute. This 1 minute period was chosen as the period for impact acceleration data extraction, and was standard for all participants. Participants were encouraged to continue running beyond this time, so that they would be blinded to the specific time period of data collection. Participants ceased running when they felt comfortable to do so, provided they had ran for a minimum of 7 minutes (6 minutes familiarisation + 1 minute data collection).” | 176-181 | Apologies for the misunderstanding previously. A more in-depth description of the running protocol has been added to the methods section of the manuscript. |
| Methods | 219-220 | The reviewer is not a biostatistician. But the reviewer is asking to check those criteria and reference again to make sure that the authors define properly according to Cohen since they are referencing Cohen here. | “[trivial effect size = 0.00 - 0.19; small effect size 0.20 – 0.49; moderate effect size = 0.50 – 0.79, and large effect size = ≥ 0.80].” | 219-220 | Thank you for noting this. The figures have been amended to reflect Cohen more accurately |
| Conclusion | 354-356 | The authors have answered the question. It may be better to add this explanation in the revised manuscript as well. | “These findings suggest that Rate_accel_ at the sacrum is an appropriate objective measure to distinguish recently injured runners, potentially informing rehabilitation goals for runners with higher rates of acceleration at the sacrum when returning to running following RRIs. Examples of gait re-training for impact acceleration attenuation have been observed through the literature [52–54], and have proven to be effective in reducing impact loading in both injured and uninjured populations.” | 356-359 | Thank you. An explanation has been added to the conclusion section. |
