# Supplementary material for: Comparison of impact accelerations between injury-resistant and recently injured recreational runners
Source: PLoS One. 2022 Sep 9;17(9):e0273716. doi: 10.1371/journal.pone.0273716 (PMC9462674; doi:10.1371/journal.pone.0273716)
Supplement: Attachment — Submitted filename: Reviewer Comments PONE-D-22-06813.pdf [file pone.0273716.s001.pdf]

Comparison of impact accelerations between injury-resistant and recently injured recreational runners

Reviewer Comments:

The study compared the magnitude of impact acceleration ($Peak_{accel}$) and rate of impact acceleration ($Rate_{accel}$) at the tibia and sacrum for three groups of runners: i) those who have never been injured, ii) who have injury resistance, not injured in past two years, and iii) who have been recently injured, 3-12 months ago. The authors mainly found that recently injured runners have greater $Rate_{accel}$ than never injured and acquired injury resistant runners.

The reviewer has the following concerns:

1) The reviewer understands how authors categorized into three groups. Based on the findings, how would the authors translate the results for the runners who had injuries in the past within 1-2 years? I mean, this group is missing in the analyses. Is there any specific reason why this group has not been included in the analyses?
2) Lines 84-86: Authors stated, "However, impact accelerations at the sacrum have rarely been assessed despite the prevalence of lower back and hip injuries experienced in runners." Why is that?
3) Line 140: Take out the line "Participants completed a Physical Activity Readiness Questionnaire (PAR-Q)" unless the authors report anything from this questionnaire, at least in this manuscript.
4) Lines 162-163: What was the actual treadmill running time? The authors stated 6 minutes but mentioned that this was familiarization with treadmill running. Why were specific 6 minutes chosen for familiarization?
5) Lines 170-171: $Peak_{accel}$ was taken from the initial contact. Is it for both the tibia and sacrum? Why was initial contact chosen to identify the peak value?
6) Figure 1 legend is confusing. Do the left and right figures mean for shank and sacrum? Then please fix (A) and (B) explanations accordingly.
7) Lines 188-190: Give some references to remove the outliers based on the condition that the values are beyond 1.5 times of upper and lower quartiles.

8) Lines 201-202: Please mention the specific references based on what effect size criteria were chosen, such as trivial effect size = 0-0.19, small effect size = 0.2-.59. Are these chosen based on reference 37?
9) Lines 206-207: The reviewer is unsure how 147 subjects were chosen from a total of 310 subjects. The reviewer acknowledges the criteria for selecting the group as injury types, sex, quarterly annual mileage, and typical training speed; did only these 147 subjects match these criteria above out of 310 or more? Is there any bias when selecting 49 subjects for each group?
10) Table 2 has not been explained in the Results section. The authors only stated it in lines 211-212. Also, table 2 says that there were 86 injuries among 49 runners. Considering the breakdown for RRI, 36*1 + 11*2 + 1*3 + 1*4 = 65. The reviewer is unsure how the authors got 86 injures for 49 runners, or the reviewer is missing something here. Clarify this.
11) Discussion: 1st paragraph, lines 250-285, is a long one. Divide into multiple sections.
12) Please include hypothesis 1 in line 254. During the last paragraph of the introduction, the authors mentioned their primary hypothesis, but nothing was mentioned in the discussion. Include hypothesis around line 254, where authors stated the primary findings, then go for the discussion based on the findings and relate the findings to the hypothesis.
13) Conclusion: Lines 330-342: I would concise the two paragraphs into one paragraph and make it a little short.
14) Since the authors main findings suggest that $Rate_{accel}$ at the sacrum was higher in recently injured runners. Does this mean that they are more likely to be injured than the other two groups? If not, please explain why? If yes, the reviewer is curious about the potential solutions/rehabilitation thoughts the authors would likely suggest for recently injured runners based on that they have greater $Rate_{accel}$ at the sacrum.
15) Lines 338-342: The reviewer is unclear about what the authors want to say here. Please rephrase. The terms ‘shock absorption’ and ‘gait re-education’ have not been discussed in the manuscript and are suddenly thrown in conclusion to end the manuscript.
