# Supplementary material for: Comparison of impact accelerations between injury-resistant and recently injured recreational runners
Source: PLoS One. 2022 Sep 9;17(9):e0273716. doi: 10.1371/journal.pone.0273716 (PMC9462674; doi:10.1371/journal.pone.0273716)
Supplement: Attachment — Submitted filename: Reviewer Comments PONE-D-22-06813_R1.pdf [file pone.0273716.s003.pdf]

Comparison of impact accelerations between injury-resistant and recently injured recreational runners

The reviewer would like to thank the authors for taking the comments. The quality of the manuscript has improved. The reviewer has the following concerns:

1) Original manuscript, Lines 84-86: Authors have answered the question regarding their views. It would be beneficial to add their thoughts to the revised manuscript.
2) Revised manuscript, Lines 220-221: This is still not clear what are the protocols for actual treadmill running. The authors added the lines in the Results section instead of the methods section without describing the protocol.
3) Revised manuscript, Lines 209-211: The reviewer is not a biostatistician. But the reviewer is asking to check those criteria and reference again to make sure that the authors define properly according to Cohen since they are referencing Cohen here.
4) The reviewer's previous comments no. 14 from the original manuscript. The authors have answered the question. It may be better to add this explanation in the revised manuscript as well.
